# Prognostic Value of Gut Microbiome for Conversion from Mild Cognitive Impairment to Alzheimer’s Disease Dementia within 4 Years: Results from the AlzBiom Study

**DOI:** 10.3390/ijms25031906

**Published:** 2024-02-05

**Authors:** Christoph Laske, Stephan Müller, Matthias H. J. Munk, Iris Honold, Matthias Willmann, Silke Peter, Ulrich Schoppmeier

**Affiliations:** 1 Section for Dementia Research, Hertie Institute for Clinical Brain Research, Department of Psychiatry and Psychotherapy, University of Tübingen, 72076 Tübingen, Germany; 2 Department of Psychiatry and Psychotherapy, University of Tübingen, 72076 Tübingen, Germany; stephan.mueller@med.uni-tuebingen.de (S.M.); matthias.munk@med.uni-tuebingen.de (M.H.J.M.); iris.honold@med.uni-tuebingen.de (I.H.); 3 German Center for Neurodegenerative Diseases (DZNE), 72076 Tübingen, Germany; 4 Department of Biology, Technische Universität Darmstadt, 64277 Darmstadt, Germany; 5 SYNLAB MVZ Leinfelden-Echterdingen GmbH, Labor Dr. Bayer, 70771 Leinfelden-Echterdingen, Germany; will80@gmx.de; 6 Institute of Medical Microbiology and Hygiene, University of Tübingen, 72076 Tübingen, Germany; silke.peter@med.uni-tuebingen.de

**Keywords:** Alzheimer’s disease, intestinal microbiome, taxonomic data, functional data, ensemble learning

## Abstract

Alterations in the gut microbiome are associated with the pathogenesis of Alzheimer’s disease (AD) and can be used as a diagnostic measure. However, longitudinal data of the gut microbiome and knowledge about its prognostic significance for the development and progression of AD are limited. The aim of the present study was to develop a reliable predictive model based on gut microbiome data for AD development. In this longitudinal study, we investigated the intestinal microbiome in 49 mild cognitive impairment (MCI) patients over a mean (SD) follow-up of 3.7 (0.6) years, using shotgun metagenomics. At the end of the 4-year follow-up (4yFU), 27 MCI patients converted to AD dementia and 22 MCI patients remained stable. The best taxonomic model for the discrimination of AD dementia converters from stable MCI patients included 24 genera, yielding an area under the receiver operating characteristic curve (AUROC) of 0.87 at BL, 0.92 at 1yFU and 0.95 at 4yFU. The best models with functional data were obtained via analyzing 25 GO (Gene Ontology) features with an AUROC of 0.87 at BL, 0.85 at 1yFU and 0.81 at 4yFU and 33 KO [Kyoto Encyclopedia of Genes and Genomes (KEGG) ortholog] features with an AUROC of 0.79 at BL, 0.88 at 1yFU and 0.82 at 4yFU. Using ensemble learning for these three models, including a clinical model with the four parameters of age, gender, body mass index (BMI) and Apolipoprotein E (ApoE) genotype, yielded an AUROC of 0.96 at BL, 0.96 at 1yFU and 0.97 at 4yFU. In conclusion, we identified novel and timely stable gut microbiome algorithms that accurately predict progression to AD dementia in individuals with MCI over a 4yFU period.

## 1. Introduction

Mild cognitive impairment (MCI) is a common syndrome in elderly people, representing an intermediate clinical stage between the expected cognitive decline of normal aging and early features of dementia [1]. Longitudinal studies provide evidence for different patterns of progression of MCI patients, ranging from the development of Alzheimer’s disease (AD) dementia to the stabilization or even reversion of cognitive impairment [2]. In clinical practice, a combination of clinical, neuropsychological, biochemical and multimodal neuroimaging findings offers, at the MCI stage, substantial information on underlying pathologies, allowing for an early recognition of prodromal AD individuals. Early detection of MCI individuals at high risk for conversion to AD dementia is crucial for administering targeted early intervention and preventing cognitive decline. AD is the most common cause of dementia in the elderly. The neuropathology of AD is characterized by the accumulation of β amyloid (Aβ) plaques and tau-containing neurofibrillary tangles (NFTs), cortical atrophy and neuroinflammation including altered microglia function in the brain [3]. In particular, the mechanisms triggering these inflammatory changes in the brain are elusive.

A growing body of evidence suggests that the gut microbiome is linked to the pathogenesis of AD. Studies examining several animal models of AD have found altered gut microbiome composition—even before the presence of amyloid plaques in the brain and thus already at a very early stage of AD pathogenesis [4]. Gut bacteria seem to influence the development of AD pathology in the brain. Germ-free animal models of AD develop less amyloid and tau pathology in the brain compared to animal models of AD with an existing or replaced intestinal microbiome [5,6,7,8]. In addition, the transfer of healthy intestinal microbiota reduces plaque and the tangle load, ameliorates reactive glia and improves cognition [9]. These findings indicate that restoring gut microbial homeostasis may have beneficial effects on AD treatment. Human studies have also demonstrated altered gut microbiome composition in AD patients [10,11,12,13,14], in MCI patients [11,12] and even in individuals with preclinical AD [15]. Some of these studies indicate that the gut microbiome could be used as a diagnostic biomarker for the detection of AD patients [11,12,13,14,15]. However, longitudinal data of the gut microbiome and knowledge about its prognostic significance for the development and progression of AD are limited.

The aim of the present longitudinal study was to examine the predictive power of taxonomic and functional intestinal microbiome data and their combination with clinical data for conversion from MCI to AD dementia within 4yFU.

## 2. Results

### 2.1. Discriminatory Ability of the Gut Microbiome between Stable MCI Patients and AD Dementia Converters

The clinical and demographic characteristics of the study sample are presented in Table 1, showing no significant differences in the clinical and demographic characteristics at baseline between stable MCI patients and AD dementia converters. As expected, AD dementia converters showed significantly lower MMSE values at 1yFU (*p* = 0.0108) and 4yFU (*p* < 0.0001) compared with stable MCI patients.

**Figure 1:**
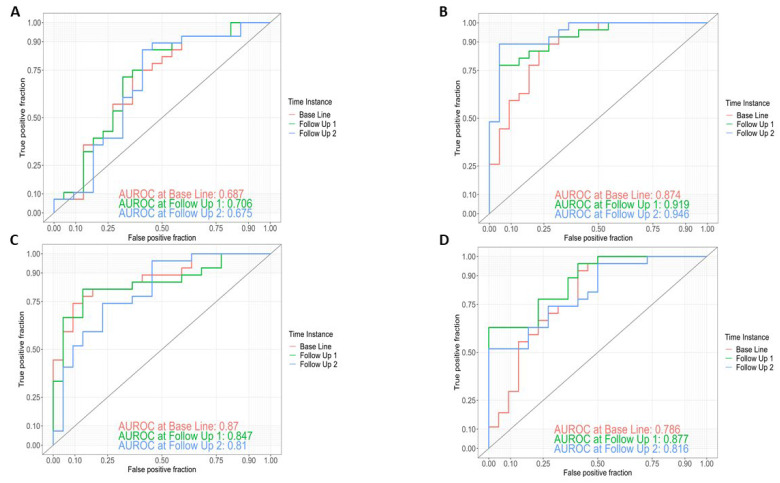
(**A**–**D**): ROC curves for discrimination between AD dementia converters (*n* = 27) and stable MCI patients (*n* = 22) within a follow-up of 4 years at baseline, at 1yFU (Follow Up 1) and 4yFU (Follow Up 2) based on (**A**) a clinical model with 4 features (age, gender, BMI, ApoE); (**B**) a genera model with 24 features; (**C**) a GO model with 25 features; and (**D**) a KO model with 33 features.

In order to evaluate whether the clinical or gut microbiome parameters were valid to predict conversion from MCI to AD dementia, we determined different logistic regression models and investigated their discriminatory potential using ROC analysis. The clinical model including age, gender, BMI and ApoE genotype yielded an area under the receiver operating characteristic curve (AUROC) of 0.69 at BL, 0.71 at 1yFU and 0.68 at 4yFU (Figure 1A).

The best taxonomic model for the discrimination of AD dementia converters from stable MCI patients included 24 genera, yielding an AUROC of 0.87 at BL, 0.92 at 1yFU and 0.95 at 4yFU (Figure 1B). The 24 genera included in the genera model are listed in Table 2.

The best models with functional data were obtained via analyzing 25 GO (Gene Ontology) features with an AUROC of 0.87 at BL, 0.85 at 1yFU and 0.81 at 4yFU (Figure 1C) and 33 KO (Kyoto Encyclopedia of Genes and Genomes [KEGG] ortholog) features with an AUROC of 0.79 at BL, 0.88 at 1yFU and 0.82 at 4yFU (Figure 1D).

The 33 KO features included in the KO model are listed in Table 3, and the 25 features included in the GO model are listed in Table 4.

**Figure 2:**
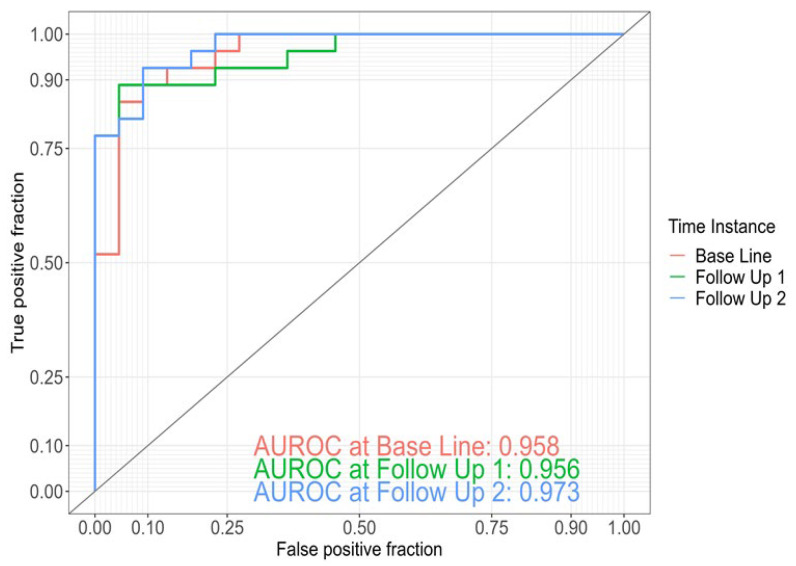
ROC curves for discrimination between AD dementia converters (*n* = 27) and stable MCI patients (*n* = 22) within a follow-up of 4 years at baseline, at 1yFU (Follow Up 1) and 4yFU (Follow Up 2) based on ensemble learning including genera model, GO model, KO model and a clinical model with 4 features (age, gender, BMI, ApoE).

Using ensemble learning for these three models, including a clinical model with the four parameters of age, gender, body mass index (BMI) and Apolipoprotein E (ApoE) genotype, yielded an AUROC of 0.96 at BL, 0.96 at 1yFU and 0.97 at 4yFU (Figure 2).

### 2.2. Longitudinal Development of Gut Microbiome

**Figure 3:**
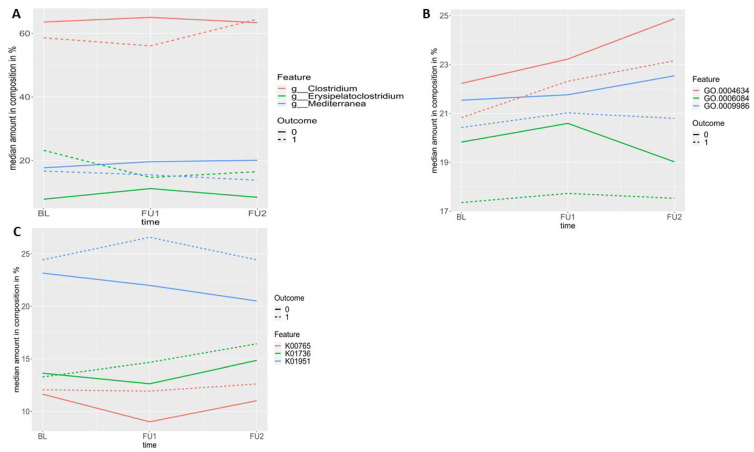
(**A**–**C**): Spaghetti plots of the three most abundant features in AD dementia converters (*n* = 27) and stable MCI patients (*n* = 22) within a follow-up of 4 years at baseline, at 1yFU (FU1) and 4yFU (FU2) in the (**A**) genera model; (**B**) GO model; and (**C**) KO model.

The longitudinal development (baseline, 1yFU, 4yFU) of the three most abundant features included in the models is depicted in Figure 3A–C. Within the genera model, including 24 features, the three most abundant genera in all participants at baseline are *Clostridium* (59.6%), *Mediterranea* (17.2%) and *Erysipelatoclostridium* (14.7%), accounting for 91.5% of all genera at baseline (Figure 3A).

Within the GO model, including 25 features, the three most abundant features in all participants at baseline are Phosphopyruvate hydratase activity (GO.0004634; 21.6%), cell surface binding (GO.0009986; 21.1%) and Acetyl-CoA metabolic process (GO.0006084; 19.4%), accounting for 62.1% of all features at baseline (Figure 3B).

Within the KO model, including 33 features, the three most abundant features in all participants at baseline are Guanosine monophosphate (GMP) synthase (K01951; 23.4%), Chorismate synthase (K01736; 13.5%) and ATP phosphoribosyltransferase (K00765; 12.0%), accounting for 48.9% of all features at baseline (Figure 3C).

## 3. Discussion

In the present study, we investigated the predictive power of taxonomic and functional intestinal microbiome data and their combination with clinical data for conversion from MCI to AD dementia within 4yFU. Within the taxonomic data, we identified a genera model with 24 features and within the functional data, we identified a GO model with 25 features and a KO model with 33 features showing the best results for the discrimination of AD dementia converters from stable MCI patients. These findings indicate that alterations in the bacterial taxa on a community level rather than single bacterial taxa and changes in functional networks rather than single functional parameters may be associated with AD dementia development in MCI patients.

The key idea of the present longitudinal study was to focus on the stability of model features over time, as the number of possible predictive models that could be developed for gut microbiome features is limitless and would have highly variable feature sets. This approach provided a limited set of trait biomarker candidates, which are stable over time in the examined cohort, as indicated by a stable predictive power of the models at all three time points. As a potential advantage of timely stable gut microbiome models, they could be used at a larger frame of disease development. A recent study has also identified a stable gut microbiome pattern in patients with Parkinson’s disease (PD) and in healthy controls over a period of 14 months [17].

In a next step, we compared the discriminatory ability of the different models used in the present study. We found that the taxonomic data worked comparably well like the functional data at baseline and slightly better at 1yFU and 4yFU. Any of the three examined microbiome models performed better than the clinical model with the 4 parameters: age, gender, BMI and ApoE. Finally, we were interested to examine if a combination of the genera model with the two functional models and the clinical model using an ensemble-learning approach provides superior discriminability compared to the included singular models. The ensemble model performed better than any of its included models separately at all three time points. Compared to the prognostic accuracy of the clinical model alone, the additional analysis of the microbiome data increased the prognostic accuracy by about 0.3 points at all three time points, underlining that the gut microbiome represents an innovative and meaningful prognostic supplement in AD.

When looking more specifically at the identified features of the taxonomic and functional models, it is convincing that these features may enhance the prognostic accuracy for conversion from MCI to AD dementia as previous studies have already linked some of them to the pathogenesis of AD [12,14,18,19]. In our genera model, 22 of the 24 taxa (91.7%) belong to the same 4 phyla: *Pseudomonadota* (formerly synonym *Proteobacteria*), *Bacteroidota* (formerly synonym *Bacteroidetes*), *Actinomycetota* (formerly synonym *Actinobacteria*) and *Bacillota* (formerly synonym *Firmicutes*). Six of the twenty-four taxa (25.0%) in our genera model belong to the phylum *Pseudomonadota* with significantly increased levels of *Teredinibacter* in AD dementia converters. The potential meaning of *Pseudomonadota* in AD has also already been shown in our previous study, where 62.5% of taxa with higher levels in AD patients compared to the healthy controls belonged to the phylum *Pseudomonadota* [14]. In addition, a second study found increased *Pseudomonadota* levels in AD patients, correlating with the severity of cognitive impairment [12]. Six of the twenty-four taxa (25.0%) in our genera model belong to the phylum *Bacteroidota* with significantly increased levels of *Filimonas* and decreased levels of *Alkaliflexus* and *Geofilum* in AD dementia converters. *Bacteroidota* and *Pseudomonadota* are Gram-negative bacteria, and lipopolysaccharides (LPS) on their surface can induce the activation of macrophages toward a pro-inflammatory phenotype [20,21]. In line with this, *Bacteroidota* and *Pseudomonadota* have been associated with several inflammatory intestinal and extra-intestinal diseases [20,22]. Inflammatory processes including altered microglia function in the brain play an important role in the pathogenesis of AD [23,24]. Therefore, gut microbial dysbiosis with up-regulation of pro-inflammatory bacteria such as *Bacteroidota* and *Pseudomonadota* could trigger these inflammatory changes and thus enhance conversion from MCI to AD dementia. In addition, *Bacteroidota* are a major producer of propionate, a short-chain fatty acid (SCFA) in the gut [25], which has been demonstrated to induce amyloid and tau pathology in animal models of AD [8,26]. Six of the twenty-four taxa (25.0%) in our genera model belong to the phylum *Actinomycetota*. *Actinomycetota* are Gram-positive bacteria and decreased levels have been described in AD patients [10]. Furthermore, *Actinomycetota* was the most abundant bacterial phylum in postmortem AD brain samples [18]. Four of the twenty-four taxa (16.7%) in our genera model belong to the phylum *Bacillota* with significantly increased levels of *Erysipelatoclostridium* in AD dementia converters. *Erysipelatoclostridium* belongs to the phylum *Bacillota* and is involved in the absorption of polyphenolic compounds in the gut [27]. A recent study showed a lower abundance of *Erysipelatoclostridium* in AD patients compared to normal controls [19]. In addition, a higher abundance of *Erysipelatoclostridium* in the gut has been described to be associated with better cognitive function in APP/PS1 mice [28] and with lower serum levels of phosphorylated tau (pTau)181 and glial fibrillary acidic protein (GFAP) in human AD patients [29]. Our findings of increased levels of *Erysipelato clostridium* in AD dementia converters could indicate a compensatory upregulation of this potential beneficial genus in the early development of AD.

Among the 33 features included in the KO model for discrimination between AD dementia converters and stable MCI patients, there were 7 mediators (K02474, K00765, K03270, K01923, K01736, K05350, K00281) producing different metabolites such as nucleotide sugars, amino acids and lipopolysaccharides. AD dementia converters showed significantly increased levels of K02474 and K00281, associated with amino sugar and nucleotide sugar metabolism and amino acid metabolism. The KO model included six mediators (K02405, K03408, K00575, K07718, K02398, K07814) of the two-component signal transduction system, connecting the input stimuli to the biofilm formation of bacteria [30]. AD dementia converters showed decreased levels of K07814. Biofilms are defined as microbes that are encapsulated in an extracellular, self-produced, biofilm matrix consisting, e.g., of functional amyloid or amyloid-like fibers, such as the amyloid curli [31]. Studies have shown that the immune system recognizes both bacterial amyloid curli and human amyloids through the same receptors, inducing inflammatory processes [32]. Recent work indicates that curli can participate in the self-assembly process of pathological human amyloids, which might also trigger amyloid pathology in AD [33]. Also, the four mediators (K02405, K02396, K02387, K02398) of flagellar assembly have been shown to be associated with directed bacterial mobility (chemotaxis). In addition, the KO model included three ATP-binding cassette (ABC) transporters (K17235, K02041, K02065), mediating the transport of arabino-oligosaccharide (AOS), phosphonate and phospholipid/cholesterol/gamma-HCH across the cellular lipid membranes in the brain parenchyma and especially at the blood–brain barrier (BBB). In line with this finding, ABC transporters have been described as key players in AD [34].

Among the 25 features included in the GO model for discrimination between AD dementia converters and stable MCI patients, there were 5 mediators (GO.0047536, GO.0016041, GO.0010133, GO.0003842, GO.0004657) associated with glutamate metabolism. Two of these five mediators (GO.0047536 and GO.0004657) showed significantly increased levels in AD dementia converters. Glutamate is the most abundant excitatory neurotransmitter in the mammalian central nervous system (CNS) and glutamate-mediated neurotoxicity has been implicated in the pathogenesis of AD [35].

This study has potential limitations. Firstly, this is a pilot study with a limited number of samples. The promising results should be replicated in a larger longitudinal follow-up study. Secondly, the follow-up measurements were performed after one and four years, but not after two and three years. Thirdly, no cerebrospinal fluid (CSF) was available in the study participants; therefore, we could not analyze the association between the gut microbiome and AD biomarkers in the CSF.

In conclusion, we identified novel gut microbiome algorithms able to accurately predict progression to AD dementia in individuals with MCI over a 4-year follow-up. The baseline models retained their predictive power at all three time points within 4 years, indicating that the identified gut microbiome signatures for AD development are stable over time. Combining the taxonomic, functional and clinical models yielded the best discriminatory ability between the two groups. The gut microbiome represents an innovative prognostic supplement and a promising area for the identification of new targets and for developing novel interventions against AD.

## 4. Materials and Methods

### 4.1. Participants

In the present study, we investigated the intestinal microbiome in 49 MCI patients participating at the AlzBiom study over a mean (SD) follow-up of 3.7 (0.6) years (Table 1). AlzBiom is an observational longitudinal study examining the intestinal microbiome at different stages of AD and in healthy controls and is performed in the Section for Dementia Research at the Department of Psychiatry and Psychotherapy in Tübingen [14]. All participants were examined at BL, at 1yFU (1.2 ± 0.2 years) and at 4yFU (3.7 ± 0.6 years). All participants underwent mini-mental state examination (MMSE) scoring [36] and clinical assessment of cognitive status by means of the Clinical Dementia Rating (CDR) scale [37,38]. Nutrition was assessed by using a Mediterranean diet score [16]. At the end of the 4yFU, 27 MCI patients converted to AD dementia and 22 MCI patients remained stable.

Patients with MCI were recruited from the Memory Clinic of the Department of Psychiatry and Psychotherapy at the University Hospital of Tübingen. All subjects underwent diagnostic work-up for dementia including physical, neurological and psychiatric examinations as well as brain imaging. According to current criteria, patients with MCI revealed cognitive deficits (corroborated by an informant) that did not interfere with activities of daily living and the absence of dementia [39,40]. MCI patients had a global CDR score of 0.5 and reported preserved function of daily living. AD patients fulfilled the NIA-AA core clinical criteria for probable AD dementia [41], had a global CDR score of ≥1.0 and impaired function of daily living. HCs had a global CDR score of 0 and reported preserved function of daily living.

The regional ethical committee approved the study and written informed consent was obtained from each individual.

### 4.2. Determination of Apolipoprotein E (ApoE) Genotype

The procedure for determining the Apolipoprotein E (ApoE) genotype was performed as previously described [42]. The ApoE ε4 positive genotype was assigned if at least one ε4 allele was present.

### 4.3. Stool Collection, DNA Extraction and Shotgun Metagenomic Sequencing

Stool samples were collected in a sterile plastic device (Commode Specimen Collection System, Thermo Fisher Scientific, Pittsburgh, PA, USA) using the DNA/RNA Shield Fecal Collection Tube R1101 (Zymo Research, Irvine, CA, USA) and immediately sent to our laboratory via post. Samples were stored at −20 °C and DNA was extracted on the same day using ZymoBiomics DNA Miniprep Kit D4300 (Zymo Research, Irvine, CA, USA). Shotgun metagenomic sequencing was carried out at Eurofins Genomics Germany GmbH (Konstanz, Germany) using the NEBNext Ultra DNA Library kit (New England Biolabs, Ipswich, MA, USA) for DNA library preparation and an Illumina HiSeq platform for sequencing. A paired-end sequencing approach with a targeted read length of 150 bp and an insert size of 550 bp was conducted. We aimed for a median sequencing depth of 40–50 million reads per sample.

### 4.4. Metagenomic Assembly

Trimmomatic (version 0.35) was used to acquire high-quality reads through adapter removing and through a sliding window trimming [43]. Reads were trimmed to a minimum length of 100 bp. Quality control of trimmed reads was performed with FastQC version 0.11.5 (https://www.bioinformatics.babraham.ac.uk/projects/fastqc/; accessed on 6 March 2023). We used SPAdes (version 3.9.0) to assemble metagenomic scaffolds with a minimum length of 1000 bp to ensure high-quality profiling [33].

### 4.5. Taxonomic Profiling

The host removal was performed using Kraken [44]. Taxonomic profiling was performed using MetaPhlAn (Metagenomic Phylogenetic Analysis) [45]. Read counts of input samples observed at taxa levels were collected and normalized by using the rarefy function implemented in the vegan bioconductor package (version 2.6-4) [46] to compare species richness from all samples in the analysis run. For our three metagenomic datasets, we achieved a median depth of 19,407,200 reads per sample (baseline), 30,486,200 reads per sample (follow-up 1) and 28,669,500 reads per sample (follow-up 2).

### 4.6. Functional Profiling

Functional profiling was performed using HUMAnN (the HMP Unified Metabolic Analysis Network) [47]. According to OUT and Phylogenetic Investigation of Communities by Reconstruction of Unobserved States (PICRUSt) [48], we identified the functional categories based on a comparison of the Kyoto Encyclopedia of Genes and Genomes (KEGG) ortholog (KO) (https://www.genome.jp/kegg/pathway.html; accessed on 6 March 2023) and of the Gene Ontology (GO) Resource (http://geneontology.org/; accessed on 6 March 2023).

### 4.7. Statistical Analysis

The statistical software package SPSS (version 23) was used for the analysis of demographic and clinical data. For all tests, we used the threshold of *p* < 0.05 for statistical significance. Levene’s test was used to proof the homogeneity of variances. *T*-tests for independent samples were used in case of continuous variables (i.e., age and BMI). The nonparametric Mann–Whitney U-test was conducted for the analysis of the MMSE and geriatric depression scale (GDS). The Pearson chi-square test was used for gender distribution, ApoE status and medication.

As features, we investigated taxonomic data (genera), functional data (KO and GO) and clinical meta data (age, gender, BMI, ApoE). Age, gender, BMI loss and ApoE are well-established risk factors for late-onset AD [49,50] and are also influencing gut microbiome composition [51,52,53]. Therefore, we decided to use an additional clinical model including these 4 parameters. Our aim was to find a predictive model for the outcomes based on feature abundances. As taxonomic and functional profiling of data from shotgun sequencing potentially results in many features, the first aim after a normalization step was to reduce the number of features. For that, we applied an ANOVA type statistic (ATS) [54]. The calculation was performed in R using the nparLD package (version 2.2) [55]. Here, the data from all three time instances were entered in the analysis. Taking the *p*-values from this test for sieving purposes, we were able to reduce the feature count to an appropriate number of about 30 features per model. With the outcomes and after suitable renormalization, we calculated the balances of the feature compositions at baseline and trained a logistic regression model. Best baseline models (Genera, KO, GO and clinical meta data) were then applied to the data from 1yFU and 4YFU using a logistic regression approach. Receiver operating characteristic (ROC) analysis was performed to examine the discriminatory ability of the intestinal microbiome among both groups. The longitudinal development (baseline, FU1, FU2) of the three most abundant features included in all models are shown using spaghetti plots. Finally, we joined the best performing models in an ensemble-learning model, as recently described [14]. This ensemble model was trained with baseline data and subsequently applied to data from 1yFU and 4YFU.

Data analysis was performed using customized R scripts and HeidiSQL (1.3) in connection with RMariaDB (1.1.1). The model training and its feature selection was coded in R scripts relying on mlr (2.18.0) package [56]. ROC curves were calculated employing OptimalCutpoints (1.1-4) and plotted with ggplot2 (3.3.5).

## Figures and Tables

**Table 1:** Clinical and demographic characteristics of individuals with stable mild cognitive impairment (MCI) and converters from MCI to mild dementia due to Alzheimer’s disease (AD).

	Stable MCI	AD Dementia Converters	*p*-Value
Number (*n*)	22	27	
Age, years, mean (SD)	70.4 (6.9)	72.9 (7.7)	0.2492
Gender (m/f)	10/12	8/19	0.3723
MMSE at baseline, mean (SD)	26.9 (1.4)	26.9 (1.8)	0.9569
MMSE at 1-year follow-up (1yFU), mean (SD)	26.4 (1.8)	24.4 (3.2)	0.0108
MMSE at 4-year follow-up (4yFU), mean (SD)	25.9 (1.4)	20.2 (4.3)	<0.0001
GDS, mean (SD)	2.0 (1.6)	2.4 (1.8)	0.4493
Body mass index (BMI), mean (SD)	25.1 (3.8)	26.2 (4.7)	0.3715
ApoE (e4/e4 carriers/single e4 carriers/non-e4-carriers; *n*)	1/6/6	0/5/10	0.3757
Arterial hypertension (yes/no)	6/16	14/13	0.1433
Diabetes mellitus (yes/no)	0/22	3/24	0.2423
Rheumatoid arthritis (yes/no)	1/21	0/27	0.4490
NSAIDs (yes/no)	6/16	8/19	0.8559
Anticoagulants (yes/no)	0/22	0/27	n.a.
Antihypertensives (yes/no)	6/16	13/14	0.1551
Antidiabetics (yes/no)	0/22	1/26	0.9853
Statins (yes/no)	4/18	7/20	0.7325
Antidepressants (yes/no)	2/20	7/20	0.1595
AChE inhibitors (yes/no)	1/21	4/23	0.3622
Mediterranean diet score [16]	32.0 (5.1)	30.1 (5.2)	0.1141

Note: Values are expressed as mean (standard deviation). N: number; MCI: mild cognitive impairment; AD: Alzheimer’s disease patients; m/f: male/female; MMSE: mini-mental state examination; GDS: Geriatric Depression Scale; NSAIDs: nonsteroidal antiphlogistics; AChE: acetylcholinesterase.

**Table 2:** Genus and phylum of the 24 features included in the genera model for discrimination between AD dementia converters and stable MCI patients. * <0.05; ** <0.01; *** <0.001 https://www.ncbi.nlm.nih.gov/Taxonomy/Browser/wwwtax.cgi?id=2 (accessed on 9 November 2023).

Genus	Phylum	Genera Levels in AD Dementia Converters vs. Stable MCI Patients (↑ Increased, ↓ Decreased)
Clostridium	Bacillota	↓
Erysipelatoclostridium	Bacillota	↑ ***
Mediterranea	Bacteroidota	↓
Lawsonibacter	Bacillota	↓
Sebaldella	Fusobacteriota	↑ **
Haloglycomyces	Actinomycetota	↑
Actinoalloteichus	Actinomycetota	↑
Arsenicicoccus	Actinomycetota	↑
Serinicoccus	Actinomycetota	↑
Knoellia	Actinomycetota	↓
Proteiniclasticum	Bacillota	↑
Alkaliflexus	Bacteroidota	↓ *
Geofilum	Bacteroidota	↓ *
Filimonas	Bacteroidota	↑ **
Terrimonas	Bacteroidota	↓
Pricia	Bacteroidota	↓
Lautropia	Pseudomonadota	↓
Acidiphilium	Pseudomonadota	↓
Komagataeibacter	Pseudomonadota	↑
Teredinibacter	Pseudomonadota	↑ **
Methyloglobulus	Pseudomonadota	↑
Oceanococcus	Pseudomonadota	↑
Persephonella	Aquificota	↑ *
Kutzneria	Actinomycetota	↓

**Table 3:** KO (Kyoto Encyclopedia of Genes and Genomes [KEGG] ortholog) labels, names and pathways of 33 features included in the KO model for discrimination between AD dementia converters and stable MCI patients. * <0.05; ** <0.01; https://www.genome.jp/kegg/pathway.html (accessed on 9 November 2023).

KO Label	Name	Pathways/Reaction Mechanisms	Relative Abundance of KO Values in AD Dementia Converters vs. Stable MCI Patients (↑ Increased, ↓ Decreased)
K02474	UDP-N-acetyl-D-galactosamine dehydrogenase	Metabolism of amino sugar and nucleotide sugar Biosynthesis of O-Antigen nucleotide sugar Biosynthesis of nucleotide sugars	↑ *
K17235	Arabinosaccharide transport system permease protein	ATP-binding cassette (ABC) transporters	↓
K02405	RNA polymerase sigma factor for flagellar operon FliA	Two-component system Biofilm formation of pseudomonas aeruginosa, escherichia coli and vibrio cholerae Flagellar assembly	↓
K07742	Uncharacterized protein	Unclassified	↓ **
K02041	Phosphonate transport system ATP-binding protein	ABC transporters	↓
K03826	Putative acetyltransferase	Protein modification	↓ *
K03706	Transcriptional pleiotropic repressor	Senses the intracellular pool of branched-chain amino acids	↓
K01951	Guanosine monophosphate (GMP) synthase	ATP + xanthosine 5’-phosphate + L-glutamine + H_2_O ⇌ AMP + diphosphate + GMP + L-glutamate Metabolism of purine, drugs and nucleotides	↑
K02065	Phospholipid/cholesterol/gamma-HCH transport system ATP-binding protein	ABC transporters	↑
K00765	ATP phosphoribosyltransferase	Histidine metabolism Metabolic pathways Biosynthesis of secondary metabolites and amino acids	↑
K09789	Pimeloyl-[acyl-carrier protein] methyl ester esterase	Biotin metabolism Biosynthesis of cofactors	↑ *
K02654	Leader peptidase (prepilin peptidase)/N-methyltransferase	Formation of pseudopili	↓
K09766	Uncharacterized protein	Unclassified	↓
K03408	Purine-binding chemotaxis protein CheW	Two-component system Bacterial chemotaxis	↓
K03270	3-deoxy-D-manno-octulosonate 8-phosphate phosphatase (KDO 8-P phosphatase)	Biosynthesis of lipopolysaccharides and nucleotide sugars Metabolic pathways	↑
K02396	Flagellar hook-associated protein 1	Flagellar assembly	↓
K01923	Phosphoribosylaminoimidazole-succinocarboxamide synthase	Purine metabolism Biosynthesis of secondary metabolites	↑
K01736	Chorismate synthase	Biosynthesis of phenylalanine, tyrosine, tryptophan, secondary metabolites and amino acids Metabolic pathways	↑
K18928	L-lactate dehydrogenase complex protein LldE	Conversion of pyruvate to lactate and back	↑
K02387	Flagellar basal-body rod protein FlgB	Flagellar assembly	↓
K00575	Chemotaxis protein methyltransferase CheR	Two-component system Bacterial chemotaxis	↓
K07718	Two-component system, sensor histidine kinase YesM	Two-component system	↓
K06378	Stage II sporulation protein AA (anti-sigma F factor antagonist)	Regulation of DNA-templated transcription Cellular spore formation	↓ *
K06023	HPr kinase/phosphorylase	Catabolite repression in Gram-positive bacteria Phosphorylates HPr, a phosphocarrier protein of a sugar transport and phosphorylation system at a serine residue	↓
K05350	Beta-glucosidase	Metabolism of cyanoamino acid, starch and sucrose Degradation of flavonoids Biosynthesis of various plant secondary metabolites	↓
K00338	NADH-quinone oxidoreductase subunit I	Oxidative phosphorylation Metabolic pathways	↑ *
K00281	Glycine dehydrogenase	Metabolism of glycine, serine, threonine, glyoxylate, dicarboxylate, lipoic acid and carbon Biosynthesis of secondary metabolites	↑ **
K06143	Inner membrane protein	Unclassified	↑ *
K01308	Gamma-D-glutamyl-meso-diaminopimelate peptidase	Endopeptidase	↓
K13652	AraC family transcriptional regulator	Binds to the target DNA and regulates bacterial virulence by sensing small molecule inducers	↑ *
K02398	Negative regulator of flagellin synthesis FlgM	Two-component system Biofilm formation of pseudomonas aeruginosa and escherichia coli Flagellar assembly	↓
K07814	Putative two-component system response regulator	Putative two-component system response regulation	↓ *
K01277	Dipeptidyl-peptidase III	Intracellular peptide catabolism	↑

**Table 4:** GO (Gene Ontology) labels, names and pathways of 25 features included in the GO model for discrimination between AD dementia converters and stable MCI patients. * <0.05; ** <0.01; https://www.informatics.jax.org/vocab/gene_ontology/ https://amigo.geneontology.org/ (accessed on 9 November 2023).

GO Label	NAME/TERM	Definition/Reaction Mechanisms	Relative Abundance of GO Values in AD Dementia Converters vs. Stable MCI Patients (↑ Increased, ↓ Decreased)
GO.0050549	Cyclohexyl-isocyanide hydratase activity	N-cyclohexylformamide + H+ = cyclohexyl isocyanide + H_2_O	↑ **
GO.0015716	Organic phosphonate transport	Alkylphosphonate transport	↑ *
GO.0047536	2-aminoadipate transaminase activity	2-oxoglutarate + L-2-aminoadipate = 2-oxoadipate + L-glutamate	↑ **
GO.0006084	Acetyl-CoA metabolic process	Key intermediate in lipid and terpenoid biosynthesis	↓ **
GO.0006694	Steroid biosynthetic process	Formation of steroids	↑
GO.0009986	Cell surface binding	Component of the cell wall and/or plasma membrane	↓ *
GO.0016999	Antibiotic metabolic process	Antibiotic metabolism	↓ **
GO.0003935	GTP cyclohydrolase II activity	GTP + 3 H_2_O = 2,5-diamino-6-hydroxy-4-(5-phosphoribosylamino)-pyrimidine + diphosphate + formate + 3 H+	↓ *
GO.0004634	Phosphopyruvate hydratase activity	2-phospho-D-glycerate = phosphoenolpyruvate + H_2_O	↓
GO.0006399	tRNA metabolic process	tRNA metabolism	↑
GO.0016854	Racemase and epimerase activity	Configuration change in one or more chiral centers in a molecule	↑
GO.0046405	Glycerol dehydratase activity	Glycerol = 3-hydroxypropanal + H_2_O	↑
GO.0052907	23S rRNA (adenine(1618)-N(6))-methyltransferase activity	S-adenosyl-L-methionine + adenine (1618) in 23S rRNA = S-adenosyl-L-homocysteine + rRNA containing N(6)-methyladenine (1618) in 23S rRNA	↑ *
GO.0006974	DNA damage response	Cellular response to DNA damage	↑ *
GO.2000143	Negative regulation of DNA-templated transcription initiation	Limitation of DNA-templated transcription initiation	↑ *
GO.0002143	tRNA wobble position uridine thiolation	Post-transcriptional thiolation at the C2 position of an uridine residue at position 34 in the anticodon of a tRNA	↑ **
GO.0019323	Pentose catabolic process	Breakdown of a pentose, any monosaccharide with a chain of five carbon atoms in the molecule	↑ *
GO.0046104	Thymidine metabolic process	Thymidine metabolism	↑ *
GO.0016041	Glutamate synthase (ferredoxin) activity	2 L-glutamate + 2 oxidized ferredoxin = L-glutamine + 2-oxoglutarate + 2 reduced ferredoxin + 2 H+	↓
GO.0016776	Phosphotransferase activity, phosphate group as acceptor	Transfer of a phosphorus-containing group from one compound (donor) to a phosphate group (acceptor)	↑ *
GO.0033764	Steroid dehydrogenase activity, acting on the CH-OH group of donors, NAD or NADP as acceptor	Oxidation-reduction (redox) reaction in which a CH-OH group acts as a hydrogen or electron donor and reduces NAD+ or NADP, and in which one substrate is a sterol derivative	↑ *
GO.0010133	Proline catabolic process to glutamate	Proline degradation to glutamate	↑
GO.0003842	1-pyrroline-5-carboxylate dehydrogenase activity	H_2_O + L-glutamate 5-semialdehyde + NAD+ = 2 H+ + L-glutamate + NADH	↑
GO.0004657	Proline dehydrogenase activity	L-proline + acceptor = (S)-1-pyrroline-5-carboxylate + reduced acceptor (first of two enzymatic reactions in proline degradation to glutamate)	↑ *
GO.0004775	Succinate-CoA ligase (ADP-forming) activity	ATP + succinate + CoA = ADP + succinyl-CoA + phosphate	↑ **

## Data Availability

The data sets presented in this study can be found at https://www.ebi.ac.uk/ena (accessed on 30 January 24), PRJEB72198.
